# Perceptions of managers regarding prerequisites for the development of professional competence of newly graduated nurses: A qualitative study

**DOI:** 10.1111/jocn.15522

**Published:** 2020-10-21

**Authors:** Marie‐Louise Södersved Källestedt, Margareta Asp, Anna Letterstål, Margareta Widarsson

**Affiliations:** ^1^ Clinical Skills Center Centre for Clinical Research Uppsala University Västerås Sweden; ^2^ School of Health, Care and Social Welfare Mälardalen University Eskilstuna Sweden; ^3^ School of Health, Care and Social Welfare Mälardalen University Västerås Sweden; ^4^ Department of Medicine Solna Karolinska Institutet Stockholm Sweden

**Keywords:** clinical skills, education, health care, leadership, learning, patient safety, professional competence

## Abstract

**Aim and objectives:**

To describe perceptions of managers regarding prerequisites for professional competence development of newly graduated nurses following a 1‐year residency programme.

**Background:**

In general, managers are unsatisfied with the professional competence of newly graduated nurses. Therefore, they have been involved in residency programmes to support the nurses’ transition from being nursing students to professional nurses. However, perceptions of managers regarding the professional competence development of nurses have been sparingly studied.

**Design/Methods:**

Qualitative, descriptive study with a data‐driven inductive approach with content analysis to obtain an understanding of the perceptions of nine managers through interviews. EQUATOR checklist COREQ is used (see File S1).

**Results:**

Three themes emerged: (a) the nurses’ relationships with their teams and patients, (b) expectations regarding the development of practical skills and leadership skills and (c) prerequisites for continuing learning by supportive structures and a mutual responsibility between the manager and the nurse. Reflection was perceived by the managers as a cornerstone in the learning and development of professional competence. Learning theory was important, but learning practical clinical skills was essential for the nurses to develop competence and be able to perform their work, including being a leader of the team. Some structures discouraged continued learning in the development of professional competence, indicating a gap between the healthcare settings and the basic nursing programme.

**Conclusions:**

There is a gap between the university and the healthcare settings in maintaining a structure for continued learning, which requires cooperation. This gap and tension can be a driving force for the learning process of competence development. Relationships with team members and patients are considered fundamental for developing professional competence.

**Relevance to clinical practice:**

To overcome the gap between the university and the healthcare settings, the managers can facilitate nurses’ continued learning by creating structures for reflection.

**What is already known about the topic?**

- Residency programmes for newly graduated nurses are one way to support them in their transition into the nursing profession.
- Leadership style of managers can affect the performance and productivity of nurses.
- Frequent and meaningful feedback from managers is important for newly graduated nurses.

## 1 INTRODUCTION

Healthcare settings face challenges regarding recruitment and retention of nurses from an international perspective (Ahlstedt et al., 2019; Cummings et al., 2010; Ke & Stocker, 2019; Rudman et al., 2014), and residency programmes have emerged as an intervention to support newly graduated nurses (henceforth referred to as nurses) in their transition to professional nurses (Beecroft et al., 2001; Olson‐Sitki et al., 2012). However, oriented and supportive structures have a wide variation for nurses entering healthcare organisations (Kovner et al., 2007).

Nurses participating in residency programmes are highly engaged and appreciate the opportunity to meet colleagues and reflect on their experiences (Olson‐Sitki et al., 2012; Widarsson et al., 2020). Furthermore, Olson‐Sitki et al. (2012) concluded that the turnover rate decreases for nurses participating in residency programmes. The main purpose of these programmes is to continue the development of professional competence.

Nursing programmes take place at university and is performed in collaboration with a healthcare organisation, mainly in the practical part of the programme. In Sweden, the basic nursing programmes lead to a Bachelor of Science in Nursing. About 25 per cent of a programme takes place in clinical settings such as medical wards, surgical wards and primary care. The onboarding introduction programme in the current study was established by the healthcare organisation with the aim of developing nurses’ competence in leadership and a clinical gaze, as well as integrating national and local guidelines into practice by using reflection to develop critical thinking. The process includes reflection, communication, leadership and skill training.

## 2 BACKGROUND

In the current study, professional competence is defined as the capacity of an individual successfully to handle certain situations or complete a certain task or work (Eilström & Kock, 2008). Competence‐in‐use is considered a dynamic process of learning mediating between the capacity of the nurse and the requirements of the profession. This means that factors related to both the individual and the profession may facilitate or limit the extent to which the individual may use and develop professional competence. The requirements of the nursing profession are expected qualifications from a university on the one hand and from a healthcare setting on the other. However, previous research indicates that there are gaps between these requirements (Widarsson et al., 2020) and that supportive environment and institutional practice is needed (Phillips et al., 2014). Self‐understanding and self‐confidence are fundamental individual factors for competence development and understanding others. Regarding profession‐related factors, the formal and informal organisation of the workplace for workers’ participation and feedback have important implications on the kind of competence that the workers use and develop (Eilström & Kock, 2008).

The importance of nurses’ competence in relation to patient safety and quality of care has been discussed in several studies (Clarke & Aiken, 2006; Kane et al., 2007; Nilsson et al., 2014). Although nurses’ competence is important, studies have shown that managers are unsatisfied with the performance of nurses in practice, including coordinating and prioritising responsibilities and delegating tasks (Oermann et al., 2010), and this might be a reason for managers being involved in residency programmes (Olson‐Sitki et al., 2012). However, Kukkonen et al. (2020) reported that knowledge about perceptions and expectations of managers regarding the competence of nurses is limited. Therefore, the current study aimed to describe perceptions of managers regarding prerequisites for professional competence development of nurses following a 1‐year residency programme.

## 3 METHODS

### 3.1 Design

This work is part of a larger study to investigate the development of professional competence of nurses during their first year, after attending a 1‐year residency programme from the perspective of three different groups: the nurses (Reference to our study), the managers and the teachers. The current study aimed to describe the managers’ perspective using a qualitative, descriptive design with a data‐driven inductive approach (Braun & Clarke, 2006). The current study used the EQUATOR checklist Consolidated Criteria for Reporting Qualitative research (COREQ) (Tong et al., 2007).

### 3.2 Setting

The managers in the current study were involved in developing a 20‐day residency programme. The residency programme was aimed at nurses with a Bachelor of Science in Nursing as a 1‐year clinical education to develop their competence in practical skills and leadership. The specific department introduction varied between three to 12 weeks.

### 3.3 Participants and data collection

All managers (*n* = 21) having nurses (*n* = 30) working with at least 40 per cent attendance at the residency programme were invited to participate in the study. Information about the study was sent by email, and a reminder was sent within 14 days. Managers who did not answer the email after 14 days were also contacted by phone. Data were collected from managers, with previous experience of working as registered nurses. They were all women and employed in a region in the middle of Sweden. At some of the departments, the managers had shared leadership. This meant that two managers shared the leadership of the department and the staff. To enhance transferability, participants were recruited from diverse wards with different specialisations such as medicine, surgery and psychiatry involving both adults and children. Data were collected from February to March 2017. An interview guide was pilot tested and used in all interviews to enhance credibility. No corrections of the interview guide were needed. They could choose how they preferred to participate, in either an individual interview or a par interview. This option was created because some of the managers had shared leadership. Nine managers agreed to participate. To maintain confidentiality, letters were selected for each interview to indicate which quote comes from which interview (Table 1).

**Table 1:** Description of codes for each interview

Interview (*N* = 6)	Participants and capitals (*N* = 9)	Interview time (*N* = 33–59 min)
1	A,B(n=2)	59 min
2	C(n=1)	33 min
3	D,E(n=2)	49 min
4	F,G(n=2)	41 min
5	H(n=1)	49 min
6	I(n=1)	44 min

The interviews were conducted, in Swedish, by all authors, (MLSK, MA, AL, MW) at the three hospitals in the region. A semi‐structured interview guide was used with an opening question: ‘What experiences do you have regarding newly graduated nurses’ transition from formal education at the university to work life in healthcare organizations?’ The managers were then asked questions about factors that could facilitate or hinder this transition. The remaining questions covered the competence areas: integrating theory and practice, as well as competence in leadership. The interviews were audio‐recorded, lasted 33–59 min and transcribed verbatim. The later interviews did not add any further information related to the purpose of the current study. The interviews were coded to identify the quotes from the participants (Table 1).

### 3.4 Data analysis

Data were analysed using thematic analysis as described previously (Braun & Clarke, 2006). Two of the authors (MLSK and MW) followed the five phases described for the analysis and performed the analysis independently. In phase 1, the transcribed data were read several times by all authors, ensuring an overview in familiarising with the content. At this stage, notes were made regarding potentially interesting aspects. Notes, codes and themes were constructed in Swedish, according to the data. In phase 2, codes were created to organise the entire data into groups with similar meanings. In phase 3, the collecting codes were reviewed and compared by all four authors, resulting in some codes being modified and new codes being added. Potential themes and subthemes within them were identified. In phase 4, the identified themes were refined, which generated a thematic map. In phase 5, refining the essence of each theme generated a clear definition and name for the themes. To ensure the credibility of the analysis, all authors read and discussed the content several times during the analysis process. The results were then translated into English and sent to a professional language reviewer. The English and Swedish versions of the results were compared, and some discrepancies in meaning were clarified. Potential limitations in general with translation are that meanings of words can get lost in translation and not just the words, but also cultural or national differences may get lost. However, the researchers’ critical review of the translations back and forth made the result solid.

### 3.5 Ethical considerations

The study was conducted according to the Swedish Act (SFS 2003:460) (Riksdagen (Swedish parliament), 2019) concerning the Ethical Review of Research Involving Humans, also followed the principles of the Declaration of Helsinki (General Assembly of the World Medical Association, 2014). The participants did not belong to a vulnerable group, so an ethical approval from an ethical committee was not required. However, permission to conduct the study was given from the chief manager at the healthcare organisation. An assessment was made of the relationship between the value of the project and any burdens or risk which it might entail for the participants. They received both verbal and written information explaining that their participation was voluntary and that they could withdraw from the project at any time, and they provided informed consent to participate in the study and were guaranteed confidentiality.

## 4 RESULTS

The perceptions of managers regarding prerequisites for nurses’ development of professional competence are presented in three themes: ‘Building relationships’, ‘Expectations regarding skills’ and ‘Conditions for continued learning.’ Each theme has two subthemes (Table 2).

**Table 2:** Perceptions of managers regarding nurses’ development of professional competence

Themes	Subthemes
Building relationships	Building relationships in the team
	Building relationships with the patients
Expectations regarding skills	Developing practical clinical skills
	Developing leadership skills
Conditions for continued learning	Supportive structure Mutual responsibility

### 4.1 Building relationships

Building relationships with colleagues and team members as well as with patients is regarded as a prerequisite for the development of professional competence of nurses.

#### 4.1.1 Building relationships in the team

Managers perceived that they play an important part in the process of building relationships in the team by having a supportive role. A team usually comprises nurses and assistant nurses, and other occupational groups participate in the team when needed. The managers considered that their presence in the team could create trust and confidence in the nurses, which in turn would benefit the relationships. Participant I stated:

And it’s very much a question of being honest and speaking your mind. So, it’s very important that we have an atmosphere here at the ward where they feel safe and are able to speak out.

Constant feedback was reported as an important factor to develop professional competence. The managers encouraged the nurses to embrace more experienced team members’ knowledge and participate in different patient activities that they were unfamiliar with and to reflect together with the more experienced nurses. Participant I stated:

You learn by listening to others. Then of course you have to train, and you do that by speaking with a more experienced colleague.

However, there was a lack of experienced nurses, which affected the possibility of support for the development of professional competence. Consequently, the managers and other less experienced nurses and assistant nurses in the team attempted to provide support.

Some managers expressed that they did not have previous experience of reflection. Participant F stated: ‘Well, we're quite a bit behind there, it's really something … we ought to pay a lot more attention to.’

Others reported the importance of using the reflection time for expressing what has been achieved and the positive things that had happened during the shift. Participant H stated:

Yes, but we ought to do that even more. Because we are always saying that in the afternoon, we ought to gather our thoughts and say: “What were the good things we managed to do today? We need to talk about: What did we accomplish today?”

A shortcoming was reported that shared reflection within the team did not appear as a part of the daily work at the departments.

#### 4.1.2 Building relationships with the patients

Building relationships with the patients was considered a prerequisite to developing professional competence. By being close to the patient, the ability to understand and evaluate the patient's needs develops. Several factors affect this development. The managers noted that the nurses experienced challenges when executing nursing tasks while creating relationships with the patients. One explanation for this was that the nurses faced a completely different situation after graduating because they were responsible for many more patients than those in the basic nursing programme. In addition, they were responsible for performing tasks in patient care that were not specifically nursing tasks. This resulted in prioritising helping the assisting nurse, which sometimes was at the expense of their nursing tasks. According to participant D: ‘So, sure, the nurse will go out and “help out” on the floor or do other chores when there is time. But it can never be at the expense of her tasks as a nurse.’

Furthermore, the managers also stated that another obstacle for developing competence was that the patients had multiple illnesses or health conditions and needed more advanced care but the nurses did not have sufficient time needed for this care, which was also seen as affecting their ability to create relationships with the patients. Participant G expressed: ‘Shortage of staff, grueling working hours, lots of patients, lots of pressure, stressful. You probably feel that you can't quite cope, your job is never quite done.’

These are challenges in establishing relationships with the patient, partly due to lack of time, which results in a high pace of work.

### 4.2 Expectations regarding skills

The managers expected that nurses should have learned more practical clinical skills during the basic nursing programme. Continued practical clinical skill training was considered a prerequisite for further development of nurses’ competences. The managers also observed a lack of leadership skills. Reflection was considered a strategy in the learning process.

#### 4.2.1 Developing practical clinical skills

The nursing profession was described as a practical profession, and the managers highlighted the need for more practical skills training for the development of professional competence. Participant H expressed:

Nursing is all right, but you need to get a lot of practice at meeting sick people, and practical skills; all of a sudden, you are faced with a port‐a‐cath one night, or a central venous catheter. There’s a lot of hands‐on treatment sometimes.

To achieve this, the nurses need early specialised training in practical skills at each workplace. The managers noticed that when the nurses developed practical clinical skills, they acquired a feeling of self‐confidence, which was beneficial in developing other skills, such as communication with the patients. Participant B stated:

When I feel secure in my role as a nurse as regards drugs, drip‐feeding, and such, I will appreciate even more having knowledge about how to communicate and such. For that’s not a priority when you’re new. Then, you focus on doing.

A gap was described between what is learned regarding practical skills during education and the situations that they encounter in working life. The managers felt that nursing students did not always have up‐to‐date knowledge, which would be useful for their working life. Participant E stated:

I believe that working at the hospital, many are sort of critical toward the nursing school … But, hey, this is the reality. What you were taught there doesn’t necessarily play out … it’s not relevant today.

Furthermore, it was observed that the nursing school had focused too much on the theoretical reference frame, which was not required in clinical practice. According to participant G:

I don’t care about those theories. We have our way of working and being mindful of certain things … ours is still quite a special operation … Nursing school is a world of its own and the reality is different.

Even though the managers put a lot of emphasis on practical clinical skills, they expected that the nurses should be proud of their academic education wherein they had trained in being reflective.

The learning needs were seen to differ between individuals. Participant B expressed: ‘I think it's mainly a matter of personality.’ Furthermore, the managers noted that nurses had high expectations from themselves early on in their work life to be able to perform different practical skills that usually needed repeated training. Participant D stated:

You have tried unsuccessfully to insert a needle a few times, and you ask a colleague for help. Then you look, what did they do differently? Instead of just asking them to do it for you and not observing and learning. For then, you will never learn.

Therefore, the managers encouraged the nurses to consult and observe more experienced colleagues.

#### 4.2.2 Developing leadership skills

The managers perceived that the development of leadership skills is important in the development of professional competence of nurses because they thought that it takes time to develop competence as a leader. They felt that the nurses needed to feel confident in themselves and in their role as a nurse before they could lead, distribute and prioritise essentials in their work. Participant I stated:

I think it’s a matter of experience. You have to have experienced enough to feel secure. Just like I say about things theoretical, when you’ve come far enough to comprehend it, you begin to be able to lead and assign the work. But then, of course, we are all unique and different.

The managers reported that nurses who had previous experience as assistant nurses could develop their leadership skills more rapidly.

The nurses were described as being eager to develop their leadership skills, almost like a separate process in relation to practical skills. The managers expressed that more knowledge needed to be imparted about leadership skills regarding being able to supervise patients, other students and colleagues during the basic nursing programme. The managers also noted that when nurses could use practical knowledge, they could lead and distribute the work to other team members. Participant B stated:

When you feel secure in the things you do, then is the time to start looking at other tasks and what you might delegate. I mean, you don’t delegate tasks you’re not up to yourself.

The managers emphasised reflection as a means to achieve competence as a leader. Participant I stated:

If it were up to me … the last hour and a half, sit down in a group, reflect. Get your instruction aimed at your professional role and your ability as a supervisor … when you’re new, it’s a good thing to reflect, ‘you did this very well, but here’s how I would do it.’

The managers expressed that if they were to decide, and if there was time, they would introduce reflection sessions that could help the nurses in the development of leadership skills during their continuing learning.

### 4.3 Conditions for continued learning

The managers perceived that structures for introduction to workplaces were a prerequisite for continued learning. However, a lack of supportive structure and structural differences affected the nurses’ opportunities for continued learning. Moreover, the managers carried the overall responsibility for planning the conditions for continued learning.

#### 4.3.1 Supportive structure

The managers perceived the residency programme as a supportive structure for the nurses to deepen their knowledge regarding guidelines and training for practical skills. The managers appreciated that the residency programme was centrally organised, obligatory and offered reflection, which was not so frequently offered at their respective workplaces. Participant C stated:

It’s just fabulous that (the residency program) is set right from the beginning, which is never the case if you are short of time … as a new nurse, to be able to meet other nurses and meditate, for that’s really what comes naturally during training. Then, of course, it doesn’t always look quite like that in real life. You don’t take the time for that. Meditating over, “What did we do this shift? The patient showed these symptoms, and what did we do?” Just great.

In addition to the residency programme, a specific introduction to enter the work at the departments was offered as a supportive structure, which could vary in length between the departments. Some nurses were expected to be independent after 4 weeks and others after 12. A longer introduction was considered an opportunity to become more proficient in performing nursing duties in a safe manner.

Skill training days, lectures, journal clubs and patient rounds, where knowledge was shared, were used as a part of continued learning at the department. The managers wanted as many nurses as possible to attend these training sessions. Furthermore, extended repeated scenario training was also offered, which strengthened the nurses’ skills in emergency care. Participant F explained:

And yearly, we train different scenarios for emergencies. It has really helped the nurses performing the emergency care, you’re not so apprehensive about it anymore.

Regular sessions of reflection at the department were considered meaningful but difficult to arrange with the staff because of their heavy workload. Because time for reflection was lacking during their work in the wards, the managers thought that the residency programme also offered a respite for the nurses with time to reflect. Participant F stated:

It will still provide a breather, so they may reflect and think, and I think it’s been good that way.

The managers who were experienced nurses offered continued learning through mentoring and became role models for the nurses. Participant H stated: ‘In the best of worlds, if you had plenty of nurses with 10, 12 years of job experience, they would become role models without trying.’

A challenge described was that the overall knowledge and experience had decreased because of the high turnover of nurses, which affected the number of potential role models. Participant D stated:

With an inexperienced principal, you lose this unspoken knowledge … The past 3 months, we delved into mentoring. It turned out that many of us in other positions have actually been nurses for some years and may command knowledge that is not communicated (to the new nurses).

The managers considered sharing their knowledge with the nurses as important to facilitating the nurses’ continued learning. They also expressed the importance of adapting to the learning needs of each individual. Participant C stated:

… everybody is different. How they learn … some want to take home a manual on the machine and read it at home. Some want visual training, feel, touch, and learn that way. You need some fundamental starting point, but then you try to adapt to the nurse in front of you.

Despite what was stated by the managers regarding individualisation of learning needs, this was not considered when less experienced nurses were employed in highly specialised departments, as expressed by participant C:

In ordinary cases, we don’t use new nurses in our ward. Ideally, we want them to get some experience first since our work is kind of special. But due to the nurse shortage, we have been forced to make exceptions.

This was explained by the shortage of nurses. Consequently, the nurses did not receive optimal conditions as supportive structures for developing their professional competence.

#### 4.3.2 Mutual responsibility

The managers acknowledged their overall responsibility for planning nurses’ continued learning for them to develop their professional competence. Different kinds of knowledge were considered useful for the workplace and not only for the individual. However, overall, for the entire workforce at the department, the managers had the responsibility to select content for the continued learning to ensure that not everyone attended the same courses. Participant C stated: ‘It's also a matter of doing inventory as an organizer. Training is fine, but our work will not benefit from 15 nurses doing the same course on pain alleviation, even if knowledge is fine … What is our need here, really?’.

On the other hand, the managers also emphasised the nurse's responsibility to plan for further competence development. The managers also noted that the nurses wanted to rely on their knowledge acquired through participating in various courses to improve competence. Participant C stated:

I want all my nurses to join any course they apply for … I’m thrilled when someone says, “Hey, I read about this course about pain alleviation.” I will want her to join. Then, I look at the work schedule … Hmm! It might not quite work out. The price for her joining this course will be that her workmates will have to exert themselves to make it work out.

If the nurses needed support with planning for continued training and courses, they had to ask for it, as expressed by participant E: ‘There will be a lot of individual adjustment, what the person wants herself.’

The managers meant that the nurses themselves carried the responsibility to request more experienced colleagues for help in decision‐making in patient care when needed.

Nurses participating in a course were also responsible for sharing the new knowledge with other staff members in the workplace. Accordingly, there was a mutual responsibility between the managers and the nurses for continued learning.

## 5 DISCUSSION

The perceptions of managers regarding prerequisites for how nurses develop professional competence are presented in three themes: (a) the importance of relationships with their teams and patients, (b) expectations regarding the development of practical skills and leadership skills, and (c) prerequisites for continuing learning by supportive structures and a mutual responsibility between the manager and the nurse. These themes can be related to the definition of competence development, which is considered a dynamic process of learning, mediating between the capacity of the nurse and the requirements of the profession (Eilström & Kock, 2008; Widarsson et al., 2020) and that it is the competence that is used which develops further.

A dynamic process of learning can be supported by good relationships with team members, experienced nurses and managers. The managers perceived building relationships with team members and patients as a prerequisite for developing professional competence of nurses. In the current study, relationships are referred to as social capital, a factor that affects the ability to interact, learn, and acquire job satisfaction (Strömgren et al., 2016). The managers stated that they wanted to be supportive and provide good conditions for creating relationships in the team, but they were vague in expressing how they could achieve this.

Previous research showed that nurses value managers who can empower them to act on their expert judgment and provide frequent and meaningful feedback, and that the relationship with the manager is a factor for job satisfaction (Force, 2005; Olson‐Sitki et al., 2012). Price et al. (2018) highlighted relationships as a part of managers’ daily work and reported that nurses considered the effect of positive management relationships on job satisfaction and the provision of quality patient care to be important. The role of team members in competence development has been described in several studies (Ahlstedt et al., 2019; Huston et al., 2018; Leggat, 2007). Our findings highlight the importance of the relationships of nurses with patients for the learning process. The managers are responsible for organising the daily work so that the nurses can establish such relationships and improve their competence. The managers indicated that poor staffing could affect relationships with both colleagues and patients, which can make the work situation stressful.

Reflection is a fundamental prerequisite for the dynamic process of learning, and it has been an embedded aspect in all themes of the present findings. In the residential programme, structured occasions for reflection were included, and they were appreciated by the nurses as well as the managers. However, occasions for reflection were not regularly structured and planned in the clinical settings. By reflection, it is possible to intertwine theoretical and practical knowledge based on experiences (Bengtsson, 1993; Eskilsson et al., 2015), and according to Ellström and Kock (2008), skills used in work are built on interaction and self‐confidence, which are difficult to acquire if the nurses lack experience. This highlights the need for reflection that can provide different perspectives and affect skills, which is necessary for developing professional competence. In addition, Husebø et al. (2015) emphasised the importance of allowing learners to analyse their experiences by reflecting, no matter where the learning occurs. However, achieving this can be challenging for healthcare organisations in case the competence to supervise and establish structures for continuing learning is limited and lacking due to a shortage in recruitment and retention of a sufficient number of employees. A task‐focused approach does not provide a sufficient outcome for nurses’ work (Cummings et al., 2010), which in the current study was evident from the limited reflection time at the departments. The learning environment is organised in different ways considering the structure for reflection. The environment can have an encouraging or inhibiting effect on reflection, depending on how it is organised. Important factors are a safe atmosphere, time to reflect as well as the mentors and supervisor's behaviour (Mann et al., 2009).

The mediation between the capacity of nurses and the requirements of the profession is complex. A previous study showed that nurses struggled and experienced inadequacy in developing professional competence. Furthermore, they experienced that the university and healthcare organisations had different views regarding the competencies that are important in the nursing profession and different views of the learning process (Widarsson et al., 2020). There are similar findings in a review study on newly graduated nurses’ experiences of support (Gardiner & Sheen, 2016). In the current study, wherein the managers perceived that practical skills are a shortcoming of the nurses, which affected their professional competence development. The managers perceived that theory and practical skills are separate parts of the nurses’ education, and only once the nurses have developed confidence in their practical skills can they develop other skills such as leadership skills. In addition, Bisholt (2012) and Brown and Crookes (2016) have described that nurses feel insecure in the execution of practical actions. Moreover, the majority of nurses need continued practical training (Ewertsson et al., 2015). According to Kolb (1984), learning practical skills integrates both theory and practice when long‐term learning is to be achieved.

The nursing profession requirements of the university are regarded as focusing on theory and as not relevant for the ‘reality’. The perceived tension between the university and the healthcare settings will probably remain, but it is possible that this tension or gap can withdraw to competence development if one considers the tension as a dialectic process of learning (Rescher, 2007). The first stage in the process, named initiation, is a situation of positing, declaration and inauguration. In the second stage, named response, a counterreaction, reply, opposition or destabilisation is developed followed by the third stage, named revision, and readjustment. This stage is characterised by operational modification, sophistication and complexification. The third stage is a synthesis of the former stages, which means a deeper understanding at another level. The third stage forms a new initiation and the learning process continues. With this idea in mind, it is possible to regard the tension between and different views from the university and the healthcare settings as complementary, and the different emphases on the requirements of the nursing profession can be a driving force for the learning process and development. Both perspectives are needed. Only focusing on developing skills according to a role model can be an effective learning strategy, but to develop competences that will empower nurses to engage in developmental work, innovation and continuous improvements in the healthcare settings, nurses need to use competences that they have developed and are requirements of the profession according to the university. However, the university and the healthcare settings need to respect each other's perspectives despite different opinions. This can form a solid basis for collaboration and coproduction between the university and the healthcare settings for nurses’ continuing learning.

According to the definition of competence development, earlier described in present study, it is only the competence‐in‐use that will be developed (Eilström & Kock, 2008). In the study by Widarsson et al. (2020), nurses expressed that they could not stay close to the patient in order to create relations, which limited their competence development. Clear structures are needed for the different aspects of nurses’ professional development for which the university and healthcare organisations are responsible.

The managers with long clinical experience described becoming role models through mentoring nurses during their continued learning.

This finding is in line with other studies who reported that managers can be role models to nurses and the manager's leadership style can affect the nurse's performance and productivity (Dehghan Nayeri et al., 2006; Kara & Senturk, 2019; Tegelberg et al., 2019).

However, the lack of clear role models may lead to general and unclear leadership with poor relationships within the team (Wallo et al., 2013).

Gardiner and Sheen (2016) describe that experienced nursing supervisors influence the everyday care provided by new graduates more than any other person. There is a need for structural support, which should naturally include educators and mentorship. Furthermore, it is important to have a structure for mentoring and reflection sessions where evidence‐based knowledge could be shared and discussed. Tegelberg et al. (2019) described that managers became role models but that they focused on the medical perspective. To overcome this, structured evidence‐based practice nurse mentor training programmes can be used and improve job satisfaction (Pasila et al., 2017; Spiva et al., 2017; Thandar Aung & Ain Binti Jamal, 2018).

Surprisingly, the managers did not mention how they wanted to create supportive structures for the nurses to acquire professional competence. They unconsciously placed the responsibility for evolving leadership skills and finding structure for continued learning on the nurses. Mutual responsibility for continued learning lack supportive structures. The managers in the current study did not mention an individual competency plan as a part of the manager's responsibility for the continued learning of each nurse. Although they stated that they were mainly responsible for planning the continued learning, they expressed that if they were to decide, they would introduce reflection time in their departments as a supportive structure.

## 6 LIMITATIONS AND STRENGTHS

In the current study, the interviews are made both individually and in pair. Our perception is that there are no limitations in the results of these two forms of interviews. Instead, it was a strength that the two managers from the same department could discuss their topics together, since they were well‐known to each other. A strength of the current study is that the participants represent different medical specialisations.

A limitation was the small sample size. Though all managers involved in the residency programme were invited only nine accepted to participate despite numerous reminders. One explanation for this was time constraints. Regardless of the small sample size, the data material was rich.

The trustworthiness of the results is limited because no deeper information of the participants’ characteristics was collected, even though the information given was that they are women and they were from different medical specialisations in the healthcare settings. However, the trustworthiness was improved when using the same interview guide and the emerging themes and subthemes were discussed by all authors.

Since this is a qualitative study, more studies in different locations are needed to reveal the extent to which the results are transferable to other settings with similar organisational and educational systems.

## 7 CONCLUSION

Residency programmes facilitate nurses in their transition to professional nursing. There is a gap between the university and healthcare organisation in maintaining a structure for continued learning, which requires cooperation. To overcome this gap, we suggest that the managers can facilitate nurses continued learning by taking responsibility for offering the time needed to implement reflection sessions and individual planning. This gap and tension can be a driving force for the learning process of competence development. Furthermore, the managers need to realise their importance for creating supportive structures in nurses’ competency development of practical and leadership skills. They can also be role models in mentoring the nurses. A prerequisite for nurses’ development of professional competence is having relationships in the team and with the patients.

## 8 RELEVANCE TO CLINICAL PRACTICE

To overcome the gap between the university and the healthcare settings, we suggest that the managers facilitate nurses continued learning by creating structures for reflection. Further, highlight the importance of mentorship. If managers with more experience in nursing could be involved in mentoring newly graduated nurses, reflection and continued learning would be a part of the nurses everyday learning. Our study highlights the managers’ responsibility on different levels in the healthcare organisation to create structures for reflection and continued learning.

## CONFLICT OF INTEREST

The authors declare no conflicts of interest.

## AUTHOR CONTRIBUTION

The first step was for all authors (MLSK., MA., AL, MW.) to read the transcribed interviews several times to derive an overview of the content. Two of the authors (MLSK and MW) followed the phases described for the analysis and performed the analysis independently following Braun and Clarke (2006) analysis. The collecting codes were reviewed and compared by all four authors (MLSK., MA., AL, MW.) resulting in some codes being modified and new codes being added. Potential themes and subthemes within them were identified. To ensure the credibility of the analysis, all authors read and discussed the content several times during the analysis process. Two authors (MLSK., M.W.) drafted the initial draft and the other two authors (MA., AL.) made critical review to the draft. All authors have seen and approved the final version the manuscript.

## Supporting information

File S1
