# Supplementary material for: Perceptions of managers regarding prerequisites for the development of professional competence of newly graduated nurses: A qualitative study
Source: J Clin Nurs. 2020 Oct 21;29(23-24):4784–94. doi: 10.1111/jocn.15522 (PMC7756416; doi:10.1111/jocn.15522)
Supplement: Supplementary file 1 — File S1 [file JOCN-29-4784-s001.docx]

**COREQ (COnsolidated criteria for REporting Qualitative research) Checklist**

| **Topic** | **Item No.** | **Guide Questions/Description** | **Reported on Page No.** |
| --- | --- | --- | --- |
| **Domain 1: Research team and reflexivity** |  |  |  |
| *Personal characteristics* |  |  |  |
| Interviewer/facilitator   \|  \| \| --- \| | **1** | Which author/s conducted the interview or focus group?   \|  \| \| --- \| | **7** |
| Credentials   \|  \| \| --- \| | **2** | What were the researcher’s credentials? E.g. PhD, MD | **Title Page** |
| Occupation | **3** | What was their occupation at the time of the study? | **Title Page** |
| Gender | **4** | Was the researcher male or female? | **Title Page** Female |
| Experience and training | **5** | What experience or training did the researcher have? | **Title Page** |
| *Relationship with participants* |  |  |  |
| Relationship established | **6** | Was a relationship established prior to study commencement? | **No** |
| Participant knowledge of the interviewer | **7** | What did the participants know about the researcher? e.g. personal goals, reasons for doing the research | **None** |
| Interviewer characteristics | **8** | What characteristics were reported about the inter viewer/facilitator? e.g. Bias, assumptions, reasons and interests in the research topic | **None** |
| **Domain 2: Study design** |  |  |  |
| *Theoretical framework* |  |  |  |
| Methodological orientation and Theory | **9** | What methodological orientation was stated to underpin the study? e.g. grounded theory, discourse analysis, ethnography, phenomenology, content analysis | **7** |
| *Participant selection* |  |  |  |
| Sampling | **10** | How were participants selected? e.g. purposive, convenience, consecutive, snowball | **6-7** |
| Method of approach | **11** | How were participants approached? e.g. face-to-face, telephone, mail, email | **6** |
| Sample size | **12** | How many participants were in the study? | **7** |
| Non-participation | **13** | How many people refused to participate or dropped out? Reasons? | **6-7,24** |
| *Setting* |  |  |  |
| Setting of data collection | **14** | Where was the data collected? e.g. home, clinic, workplace | **7** |
| Presence of non-participants | **15** | Was anyone else present besides the participants and researchers? | **No** |
| Description of sample | **16** | What are the important characteristics of the sample? e.g. demographic data, date | **6** |
| *Data collection* |  |  |  |
| Interview guide | **17** | Were questions, prompts, guides provided by the authors? Was it pilot tested? | **7** |
| Repeat interviews | **18** | Were repeat inter views carried out? If yes, how many? | **No** |
| Audio/visual recording | **19** | Did the research use audio or visual recording to collect the data? | **7** |
| Field notes | **20** | Were field notes made during and/or after the inter view or focus group? | **No** |
| Duration | **21** | What was the duration of the inter views or focus group? | **7** |
| Data saturation | **22** | Was data saturation discussed? | **No** |
| Transcripts returned | **23** | Were transcripts returned to participants for comment and/or  correction? | **No** |
| **Domain 3: analysis and findings** |  |  |  |
| *Data analysis* |  |  |  |
| Number of data coders | **24** | How many data coders coded the data? | **7-8** |
| Description of the coding tree | **25** | Did authors provide a description of the coding tree? | **7-8** |
| Derivation of themes | **26** | Were themes identified in advance or derived from the data? | **7-8** |
| Software | **27** | What software, if applicable, was used to manage the data? | **None** |
| Participant checking | **28** | Did participants provide feedback on the findings? | **No** |
| *Reporting* |  |  |  |
| Quotations presented | **29** | Were participant quotations presented to illustrate the themes/findings? Was each quotation identified? e.g. participant number | **9-18** |
| Data and findings consistent | **30** | Was there consistency between the data presented and the findings? | **Yes** |
| Clarity of major themes | **31** | Were major themes clearly presented in the findings? | **9-18** |
| Clarity of minor themes | **32** | Is there a description of diverse cases or discussion of minor themes? | **No** |

Developed from: Tong A, Sainsbury P, Craig J. Consolidated criteria for reporting qualitative research (COREQ): a 32-item checklist for interviews and focus groups. *International Journal for Quality in Health Care*. 2007. Volume 19, Number 6: pp. 349 – 357
